# Chronic Stress Induces Retinal Ganglion Cell Degeneration Featuring Reduced Density, Altered Intrinsic Electrophysiology, and Light Responses

**DOI:** 10.3390/biology15171446

**Published:** 2026-08-24

**Authors:** Manfei Huo, Meizhen Zhu, Yuqing Wu, Zeyuan Ding, Siqi Li, Yanli Ran

**Affiliations:** 1 Key Laboratory of Preclinical Study for New Drugs of Gansu Province, Institute of Physiology, School of Basic Medical Sciences, Lanzhou University, Lanzhou 730000, China; 2 The School of Life Sciences, Lanzhou University, Lanzhou 730000, China

**Keywords:** depression, blood perfusion, retinal ganglion cells, intrinsic properties, light responses

## Abstract

Depression is often associated with functional disturbances in the visual system. However, the fundamental features underlying these visual system aberrations in depression remain to be fully elucidated. In particular, in the first stage of visual processing, how different retinal output neuron types change their intrinsic properties and output features in response to specific light stimulation remains unclear. Here, by adopting a mouse model of depression induced by chronic unpredictable stress (CUS), we found that depression is associated with reduced blood perfusion in the retinal inner plexiform layer (IPL). The hypoperfusion in the IPL is paralleled by a remarkable reduction of retinal ganglion cell (RGC) density, with more cell loss in ipRGCs than in the general RGCs. The surviving RGCs—particularly, ipRGCs—changed their intrinsic electrical properties, exhibiting decreased membrane input resistance, more depolarized resting membrane potential, and altered spiking properties. Additionally, these cells showed stimulus-size-dependent increases in light-evoked responses. Together, our findings demonstrate that depression is associated with the retinal IPL hypoperfusion and RGC impairments (particularly ipRGCs) and suggest retinal layer- and RGC-type-specific susceptibilities, furthering our understanding of retinal pathophysiology in depression.

## Simple Summary

Depression is known to be accompanied by a range of retinal pathological processes and can negatively affect retinal ganglion cells, potentially further exacerbating the disturbances in circadian rhythm and mood regulation. This study attempted to investigate how the properties of retinal ganglion cells are altered in the context of depression. In a mouse model of chronic stress-induced depression, blood perfusion was selectively reduced in the inner plexiform layer of the retina. In parallel, retinal ganglion cell density was markedly decreased, with intrinsically photosensitive retinal ganglion cells—key regulators of circadian rhythm and mood—showing significantly greater loss than the general RGC population. Surviving retinal ganglion cells, such as sustained On and transient Off alpha ganglion cells, exhibited altered intrinsic properties, characterized by reduced input resistance and a more depolarized resting membrane potential, as well as stimulus size-dependent light response changes. Together, these results reveal that depression is concomitant with retinal layer-specific hypoperfusion and type-specific retinal ganglion cell pathology, including reduced cell density, altered intrinsic properties, and altered light responses. These results provide new insights into retinal pathological features associated with depression and may help inform the development of therapeutic strategies targeting retinal cells.

## 1. Introduction

Depression is one of the most significant health challenges worldwide [1,2]. However, current treatments often have delayed therapeutic effects, undesirable side effects, and only partial efficacy (e.g., [3,4,5,6]). For efficient treatment of depression, it is critical to comprehensively understand the pathophysiology of depressive disorders. Substantial studies have indicated that the hippocampus, prefrontal cortex, amygdala, hypothalamus, and habenula are the core brain regions in depressive disorders [7,8,9,10,11]. In patients with depression and in depressive animal models, one of the most commonly observed features is circadian rhythm disruption [12,13,14,15]. One of the key circadian rhythm regulators is a type of retinal ganglion cells named intrinsically photosensitive RGCs (ipRGCs) [16,17,18]. RGCs in mammals are categorized into more than 30 types [19,20]. The ipRGCs express melanopsin that functions as a third class of photoreceptor, in addition to rods and cones [21]. These RGCs primarily mediate non-image-forming visual functions, including the regulation of melatonin secretion, circadian rhythms, and mood. In addition to ipRGCs, conventional RGCs are responsible for image-forming visual functions. These conventional RGCS-composed image-forming pathways have also been implicated in depressive disorders [22,23,24]. However, whether and how ipRGCs and general RGCs change their properties in depression remains largely unknown, particularly with regard to their respective cell numbers, physiological properties, and functional regulations.

Studies on depression indicate that a variety of pathological processes correlate with the disorder, including neuroinflammation, cardiovascular dysfunction, and impaired neurotrophic support [25,26,27,28]. These pathological processes largely affect neuronal physiology and function. In the retina, RGCs are highly sensitive to these pathological processes and may thereby suffer from damage in depression. In some retinal diseases such as glaucoma, diabetic retinopathy, and ischemia/perfusion injury, the impairments are not inhomogeneous across different retinal layers and RGC types [29,30,31,32]. In most pathological contexts, RGCs present decreased cell density and disrupted function, but ipRGCs often present higher resistance compared with general RGCs [29,30,31,32]. In depression, RGC loss has also been reported [33,34]. However, under disordered psychological conditions that rely on the regulation of ipRGCs, it remains unknown whether ipRGCs also present higher resistance than the general RGCs.

In this study, we characterized retinal pathology in a mouse model of CUS-induced depression, with a focus on blood perfusion, ipRGC and general RGC density, intrinsic properties, and light-response alterations. Our results establish a fundamental profile of the vascular and electrophysiological disturbances in ipRGCs and general RGCs in the depressive state. Importantly, these findings expand our current knowledge of depression-associated visual system dysfunction and may inform future investigations into RGC-selective therapeutic strategies.

## 2. Materials and Methods

### 2.1. Animals

C57BL/6 mice were used for all experiments throughout this study. As female mice do not present depression-like behavior after exposure to chronic stress such as was used in this study [35], we selected male mice that were 8 to 12 weeks old, with body weights ranging between 18 and 22 g. All animals were housed in the animal care facility at Lanzhou University under controlled environmental conditions, including regulated temperature, humidity, and a 12 h light-dark cycle. All animals had unrestricted access to food and water. All experiments were carried out in strict accordance with the European Community Guidelines (2010/63/EU) and the ARRIVE Guidelines. Additionally, the entire experimental protocol was reviewed and approved by the Ethics Committee of Lanzhou University (reference number: lzujcyxy20231203, 2 December 2023).

### 2.2. Chronic Unpredictable Stress

The CUS procedure used in this study was as described in a recent study [35]. In this procedure, experimental mice underwent randomized allocation to either the CUS group or the control group. Control animals were maintained under standard housing conditions, while CUS mice were exposed to a CUS paradigm and housed individually during the entire stress protocol. To maintain the unpredictable nature of the stress exposure while ensuring comparable treatment among animals, a 10-day schedule containing three stressors per day was generated using a simple custom script written in Igor Pro 9.0. For each day, the program randomly selected three stressors from a pool of validated stress paradigms. The stressors included cage tilting at 45° without bedding for 4 h, exposure to wet bedding for 24 h, continuous illumination for 24 h, crowding stress with 10 mice per cage for 2 h, short-term social isolation in a 15 cm × 15 cm isolation box for 4 h, exposure to an elevated platform for 30 min, restraint stress for 2 h, and rapid light–dark alternation at 7 min intervals for 2 h.

### 2.3. Forced Swimming Test

The forced swimming test (FST) was performed in a transparent glass cylinder measuring 25 cm in height and 10 cm in diameter. The cylinder was filled with water to a depth of 10 cm, and the water temperature was maintained at 25 ± 1 °C. Each mouse was placed in the cylinder for 6 min. Immobility was defined as the absence of active escape-directed movements, with the animal making only minimal movements required to keep its head above the water. The behavior of each mouse was video-recorded, and the immobility time during the final 4 min of the test was measured for subsequent analysis.

### 2.4. Tail Suspension Test

In the tail suspension test (TST), each mouse was fastened by adhesive tape positioned roughly 1 cm from the tail tip and suspended at a height of 50 cm above the floor. The testing period lasted for 6 min per animal. Immobility was defined as the absence of any bodily movement while the animal remained passively hanging. Any subject that exhibited tail-climbing behavior during the trial was removed from the final data set. The entire procedure was videotaped, and total immobility time was measured over the final 4 min interval of the session.

### 2.5. Optical Coherence Tomography Angiography

Optical coherence tomography angiography (isOCTA, Optoprobe, UK) was performed as previously described [32]. In brief, anaesthesia was first administered to the mice, after which pupillary dilation of both eyes was achieved via topical application of a single drop of compound tropicamide. Each animal was positioned carefully in front of the fundus camera, with the examined eye aligned close to the imaging lens and the corneal surface oriented perpendicular to the scanning beam. The optic nerve head served as the reference point for image alignment. For each eye, a coronal vascular image centered on the optic disc and a vertical scan passing through the center of the optic nerve head were obtained. Retinal vascular density was quantified at different retinal depths using VesQuant, the integrated analysis software of the OCTA system. The OCTA data were acquired from both eyes of each experimental mouse. The analyzed layers included the retinal nerve fiber layer/ganglion cell layer complex (RNFL/GCL), inner plexiform layer (IPL), and inner nuclear layer (INL). Retinal layer thickness was further measured with OctSegmentationGUI built-in segmentation software, including the inner retina (NFL + GCL + IPL), INL, outer plexiform layer (OPL), and outer nuclear layer (ONL). We utilized the built-in ReconstructionUI module (Optoprobe, UK) to automatically segment the retinal layers, based on their distinct vascular morphology, as described previously [32,36,37]. The grayscale images subjected to analysis were derived by designating the first 14 coronal frames as the NFL/GCL complex, frames 14–26 as the IPL, and frames 26–40 as the OPL. In these generated grayscale representations, vascular structures appeared as bright, high-intensity pixels. All image sets were subsequently exported to the VesQuant platform for quantitative vessel network assessment. With intensity thresholds confined to a 0–1 range, optimal skeletonization was achieved by applying tiered cutoffs: 0.6–0.75 for the superficial NFL + GCL layer, 0.5–0.65 for the intermediate IPL, and 0.3–0.55 for the deep OPL. Finally, the software performed automated calculations to yield the percentage of vessel-positive pixels, which served as our key density metric. For each layer, thickness measurements were performed within a 300-µm horizontal region beginning 200 µm away from the optic nerve head.

### 2.6. Immunohistochemistry

Mice were deeply anaesthetized via a single intraperitoneal injection of pentobarbital sodium at 100 mg/kg, followed by intracardial perfusion with 0.01 M phosphate-buffered saline (PBS; BBI, Cat No. B640011, Shanghai, China) and 4% paraformaldehyde (PFA; BBI, Cat No. E672002, Shanghai, China). Their eyes were quickly removed and the retinas were carefully isolated. Following dissection, each retina was cut into four quadrants and placed onto a black MF-Millipore™ filter membrane (0.8 μm pore size, REF: AABPO2500). The retina-filter paper assemblies were then transferred into a 24-well plate containing 4% paraformaldehyde (PFA) and left to fix for 20 min. After fixation, samples were washed six times with 0.01 M PBS, each time lasting 20 min. Subsequently, the tissues were blocked for 1 h at room temperature using a solution composed of 10% normal donkey serum and 0.5% Triton X-100 (Sigma-Aldrich, T8787, Shanghai, China). Following blocking, retinas were incubated with primary antibodies at 4 °C for 72 h. After six additional 20 min PBS washes, the samples were incubated with fluorophore-conjugated secondary antibodies for 2 h at room temperature in the dark. Finally, the retinas were washed again with 0.01 M PBS (six cycles of 20 min each) and mounted for imaging. The antibodies against the different markers included rabbit antibody to Brn3a (1:500; abcam, Cambridge, UK, ab245230) and rabbit antibody to melanopsin (1:2000; Advanced Targeting Systems, Cat: AB-N39, San Diego, CA, USA). The secondary antibody used in this study was Alexa Fluor 594-conjugated goat anti-rabbit IgG (H + L; 1:800; Invitrogen, Cat: A-11012, Shanghai, China). From each experimental mouse, one retina was used for the immunostaining of Brn3a and another one was used for the staining of melanopsin. All fluorescent images were acquired with consistent parameters and analyzed through ImageJ (version 1.51n). RGC numbers were counted automatedly using ImageJ’s particle analysis workflow in a blinded fashion. In detail, all acquired images were transformed into an 8-bit grayscale format. Then, the brightness and contrast were normalized through the software’s adjustment settings, followed by a thresholding step that converted the signal-positive regions into binary masks. Touching cellular profiles were separated via the Watershed algorithm, and the final numerical values were derived using the ‘Analyze Particles’ measurement feature. The rate of RGC loss was calculated as the difference between the mean RGC density of the control group and the RGC density of each retina from the CUS mice, divided by the mean RGC density in the control.

### 2.7. Light Stimuli for Electrophysiology

For the light stimulation, light was emitted from a Polygon1000 light source (Mightex, CA, USA) and projected onto the retina through a water-immersion objective. Two wavelengths, 505 nm and 365 nm, were chosen to match the spectral sensitivities of mouse M- and S-opsins, respectively. The light intensity was set to 2 × 10^4^ photoisomerizations (P*)/cone/s under monochromatic illumination. A small spot (diameter of 270 µm) and full-field light stimuli were used in this study. The light stimuli were set at On for 1 s followed by Off for 2 s. Each light stimulus was displayed 3 times. Prior to each recording session, the light stimulus was made concentric with the microscope’s scan field, and the patched cells were maintained at the field center to make sure the recorded neurons received the intended light stimulation.

### 2.8. Whole-Cell Patch Clamp Recordings

Mice were dark-adapted for a minimum of 2 h and subsequently euthanized by cervical dislocation under deep anesthesia with isoflurane. The dorsal side of each eyeball was marked before rapid enucleation. Enucleated eyeballs were immediately placed in carboxygenated (95% O_2_, 5% CO_2_) artificial cerebrospinal fluid (ACSF) composed of the following components (in mM): 125 NaCl, 2.5 KCl, 1 MgCl_2_·6H_2_O, 1.25 NaH_2_PO_4_, 20 glucose, 26 NaHCO_3_, 2 CaCl_2_·2H_2_O, and 0.5 L-glutamine. Continuous carboxygenation was maintained to preserve retinal viability throughout the preparation process.

After careful removal of the cornea, the sclera and lens were isolated. Then, the vitreous body was carefully cleaned from the retina, the retina was cut into 3 to 5 wings and placed ganglion cell side up on an Anodisc membrane (Cytiva, GE Healthcare, Pittsburgh, PA, USA). Subsequently, the retina, along with the Anodisc membrane, was relocated to the recording chamber of the microscope. The chamber was continuously superfused with artificial cerebrospinal fluid (ACSF) at a rate of 2 mL·min^−1^, and the temperature was held at approximately 32 °C. To visualize retinal blood vessels, 2 µL of 20 mM sulforhodamine-101 (SR101, Invitrogen/Thermo Fisher Scientific, Shanghai, China) solution was added per liter of ACSF. The entire retinal preparation procedure was conducted under red light to avoid light-induced damage.

Membrane potentials were recorded using an Axopatch 200B amplifier (Molecular Devices LLC, San Jose, CA, USA). The electrode resistance was 7–15 MΩ. The electrodes were filled with an intracellular solution containing the following (in mM): 120 K-gluconate, 5 NaCl, 10 KCl, 1 MgCl_2_·6H_2_O, 1 EGTA, 10 pure HEPES, 2 Mg-ATP, and 0.5 Tris-GTP (pH adjusted to ~7.2 with KOH). Alexa 488 dye (100–200 µM) was added to the intracellular solution to visualize the morphology of the recorded cells. As light responses of the same RGC types can vary along the dorsal–ventral retina and nasal–temporal retina [38,39,40], we recorded RGCs almost at the same retinal region, the ventral retina, relatively central along the temporal–nasal axis. To record sustained On alpha (sOn α) and transient Off alpha (tOff α) RGCs, we targeted cells with big soma (diameter ≥ 20 µm). The sOn and tOff α RGCs were subsequently confirmed based on the soma size and the light-response properties. In the whole-cell recording mode, electrophysiological recordings were acquired at a digitization rate of 10 kHz using pClamp (Molecular Devices GmbH, San Jose, CA, USA), and the raw signals were subjected to Bessel filtering with a corner frequency of 2 kHz. Then, all the electrical recorded data were analyzed using custom scripts written in IGOR Pro 9.0. Regarding the spikes generated during the injected currents, the spike numbers were counted during the current injection (500 ms). To assess spike response under light stimulation, the spike numbers were counted during the 2 s following the light onset for sOn αRGCs and following the light offset for the tOff αRGCs, respectively. Spike rates were calculated in bins of 20 ms.

### 2.9. Statistics

All statistical analyses were conducted using GraphPad Prism 10 software. The behavioral tests were performed on at least 11 mice. All imaging data for each group were acquired from at least 5 animals. The electrical data of each group were recorded from at least 4 mice. Before the statistical analysis, the data normality was checked via the Shapiro–Wilk test and the homogeneity of variances was checked using the F test. The results met the assumptions for parametric tests. Statistical comparisons between the control and CUS groups were performed using a two-tailed unpaired Student’s *t*-test. All data are expressed as mean ± standard error of the mean (SEM). Statistical significance was defined as *p* < 0.05. Significance levels are indicated as follows: * *p* < 0.05, ** *p* < 0.01, and *** *p* < 0.001.

## 3. Results

### 3.1. Chronic Stress Induced Depression and Decreased Blood Perfusion in the Inner Retinal Plexiform Layer of the Mouse Retina

**Figure 1:**
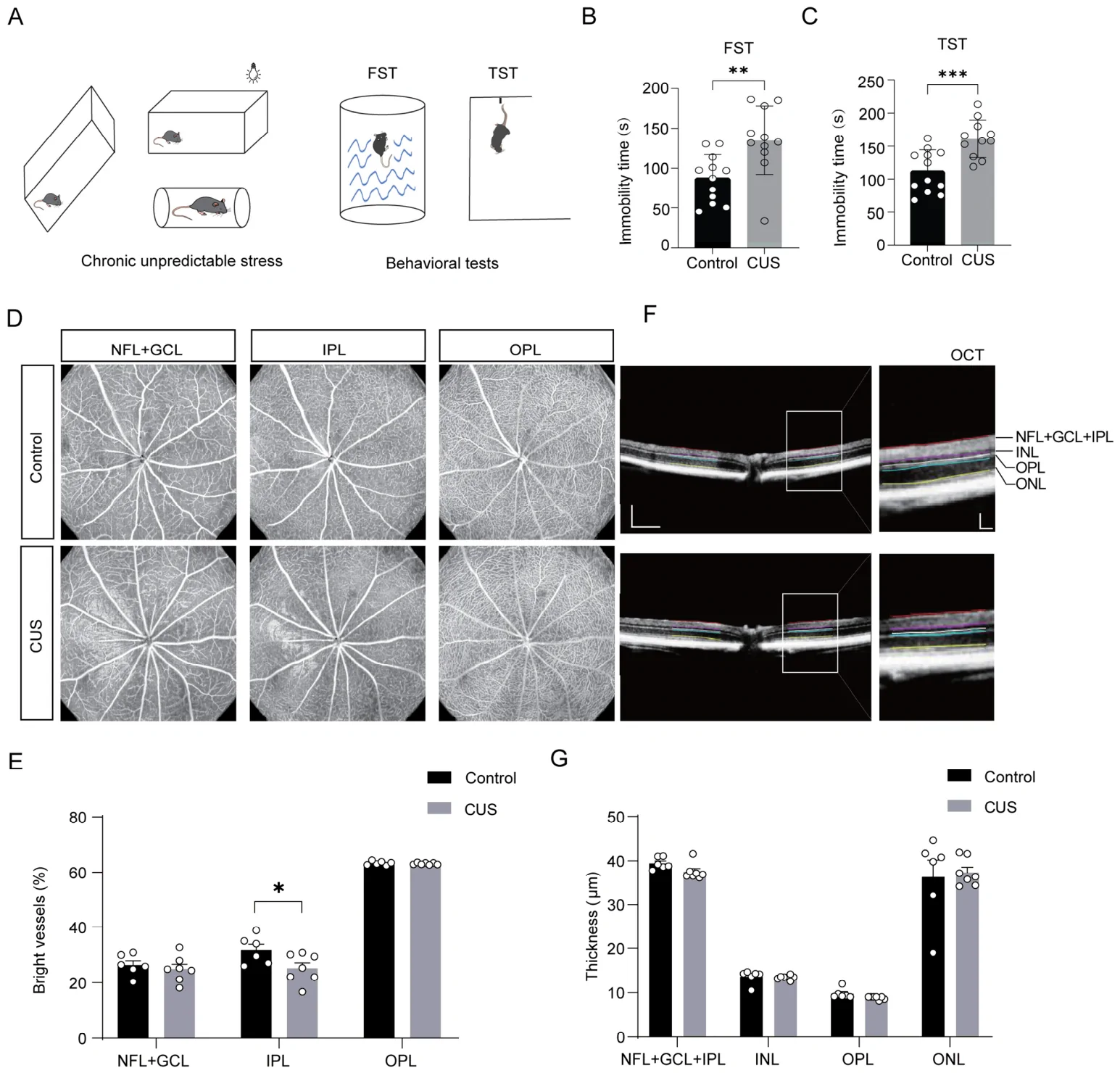
CUS induced depression-like behavior and a decrease in blood perfusion in the IPL. (**A**) Illustrating the CUS procedure and behavioral tests in mice. (**B**,**C**) The comparison of immobility time in FST and TST between normal conditioned (control: n = 12 mice) and chronically stressed mice (CUS: n = 11 mice). (**D**) Representative OCTA images illustrate the vascular networks within the NFL and GCL, IPL, and OPL. (**E**) Cross-sectional retinal tomograms derived from OCTA data, with horizontal and vertical scale bars of 250 µm and 150 µm, respectively. An enlarged view is provided to the right, where the scale bars measure 50 µm in both dimensions. (**F**) Comparison of bright vessels at different layers in the retina (NFL +  GCL, IPL, and OPL) between control (n = 6 mice) and CUS (n = 7 mice) groups. (**G**) Thickness comparison of different retinal layers between the control (n = 6 mice) and CUS (n = 7 mice) groups. Data are presented as mean ± SEM. * *p* < 0.05, ** *p* < 0.01, and *** *p* < 0.001.

To induce depression-like behavior, we adapted male mice to CUS for 10 days, as described in our previous study [35] (Figure 1A). After 10 days of CUS, we carried out FST and TST to examine whether this protocol induced depression-like behavior in the mice. We found that CUS mice showed longer immobility time in the FST and TST compared with the normal conditioned mice (Figure 1B,C), indicating that mice presented depression-like behavior after CUS.

Previous studies have shown that depression is associated with vascular pathology in the brain (e.g., decreased blood perfusion) [25,35], but whether the depression accompanied vascular comparisons is also present in the retina has not been reported. Therefore, we first compared the retinal blood perfusion based on the retina images acquired through OCTA between normal conditioned and CUS-experienced mice. By analyzing the blood vessel density based on the image brightness as in our previous study [32], we found that in the retina of stressed mice, the percentage of bright vessels in the IPL was significantly lower than that in the normal mouse retina (Figure 1D,E), indicating that the blood perfusion in the IPL was less in the CUS mice. In other retinal layers, there were no detectable differences in the bright vessels between the normal and CUS mice (Figure 1E). Regarding the thickness of different retinal layers, there were no differences between normal and CUS mice (Figure 1F,G). Thus, these data reveal that CUS induced depression-like behavior in mice and reduced blood perfusion in the retinal IPL.

### 3.2. Chronic Stress Leads to Cell Loss in Both General RGCs and ipRGCs

**Figure 2:**
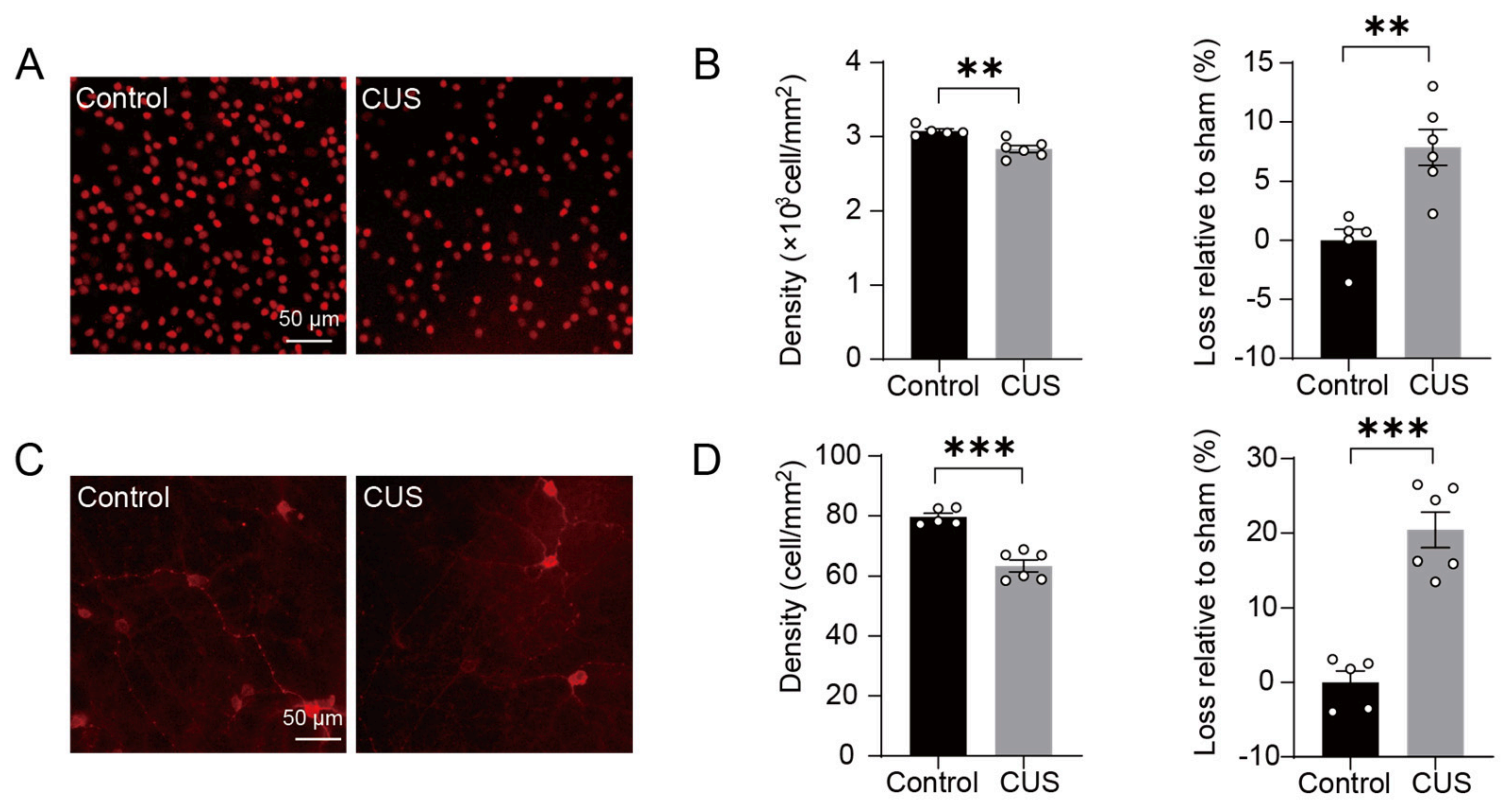
CUS leads to cell density decrease in both general RGCs and ipRGCs. (**A**) Representative images showing Brn3a^−^ positive stained cells in whole-mounted retinas from normal mice and CUS mice. (**B**) The Brn3a^+^ cell density in control and CUS mouse retina (**left**), and the relative loss of Brn3^+^ cells compared to control (**right**). (**C**) Representative images showing melanopsin-positive stained cells. (**D**) The melanopsin^+^ cell density and the relative cell loss compared to the control between the control and CUS groups. Control: n = 5 mice; CUS: n = 6 mice. Data are presented as mean ± SEM. ** *p* < 0.01, and *** *p* < 0.001.

CUS-induced retinal blood supply deficiency can lead to RGC degeneration and cell loss. While previous studies have reported that depression is associated with decreased RGC numbers, whether this occurs evenly between different RGC types remains unclear. Under many pathological conditions involving decreased retinal blood supply, ipRGCs, which play crucial roles in the regulation of mood, are more resistant than general RGCs (e.g., [29,30,31]). We examined whether ipRGCs also showed higher resistance under chronic stress. For this, we counted general RGCs and ipRGCs via immunostaining of Brn3a and melanopsin in whole-mounted retinas (Figure 2). Because the RGC loss under pathological conditions can vary between the central and peripheral retina [32], we counted the cell numbers in the middle retina to avoid the influence of retinal location on RGC loss. We found that there was significant cell loss in both general RGCs and ipRGCs (Figure 2B,D). Furthermore, we found that here, the loss percentage of ipRGCs was higher than that of general RGCs (Brn3a vs. ipRGC: 7.84 ± 1.53 vs. 20.45 ± 2.38, *p* = 0.0012), indicating that ipRGCs underwent more damage than general RGCs in depression, different from the comparatively higher resistance of ipRGCs under many other pathological conditions (e.g., [29,30,31]).

### 3.3. Chronic Stress Changes the Intrinsic Properties of sOn and tOff αRGCs

We next investigated whether and how surviving ipRGCs and general RGCs modified their intrinsic properties following CUS. To this end, intact retinal whole-mounts were prepared and maintained in vitro under dark conditions. From these, whole-cell current-clamp recordings were obtained from sOn αRGCs and tOff αRGCs. Notably, sOn αRGCs correspond to M4 ipRGCs (a type of ipRGCs), whereas tOff αRGCs belong to the general RGC population. Both subtypes are readily identifiable by their large somata and distinctive light-response properties.

**Figure 3:**
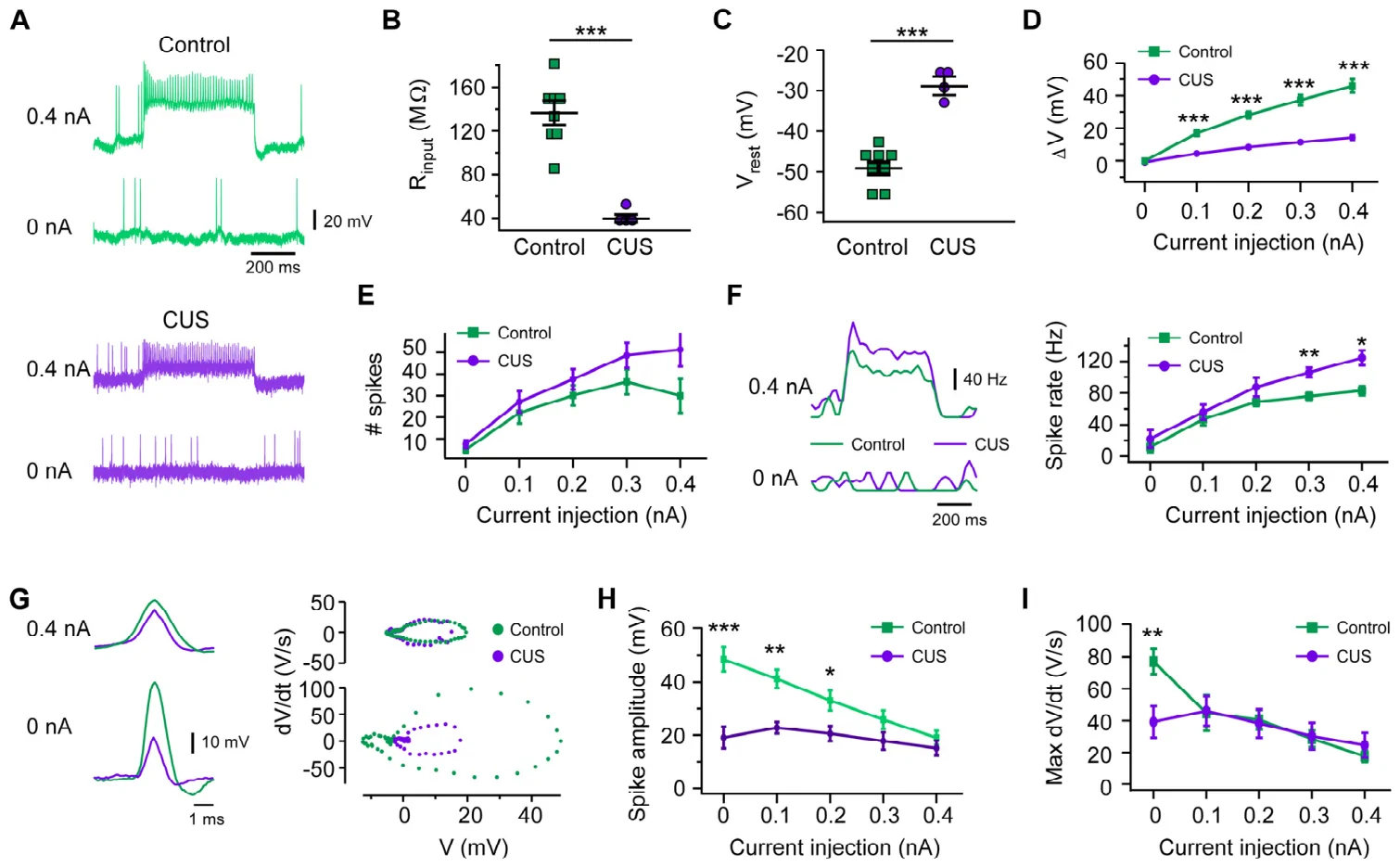
CUS induces intrinsic property alterations in sOn αRGCs. (**A**) Exemplary traces showing current injection evoked membrane potential changes for sOn αRGCs recorded from control (green) and CUS (purple) mouse retinas in darkness. (**B**,**C**) Input resistance and resting membrane potential of sOn αRGCs recorded from normal mouse and CUS mouse retinas. (**D**) The graded voltage responses of sOn αRGCs to different injected currents. (**E**) The number of spikes evoked by different injected currents. (**F**) Spike rate traces (**left**) calculated from traces in (**A**) and the maximum spike rate versus injected current (**right**). (**G**) Exemplary average spike waveforms (**left**) and their corresponding dV/dt plots (**right**). (**H**,**I**) The amplitude and maximum rising slope as a function of injected current. Control: n = 8 mice; CUS: n = 4 mice. Data are presented as mean ± SEM. * *p* < 0.05, ** *p* < 0.01, and *** *p* < 0.001.

To analyze the electric properties of sOn αRGCs and tOff αRGCs, we injected different positive currents into the patched RGCs (Figure 3A). We first calculated the passive electrical properties: input resistance and resting membrane potential. We found that the membrane input resistance of sOn αRGCs in stressed mice was much lower than that in the normal mice (Figure 3B). This lower membrane input resistance was associated with higher resting membrane potential (Figure 3C), indicating that sOn αRGCs in the CUS mice were more depolarized than those in the normal mice. Aligning with the higher resting membrane potential in the CUS mice, the injected currents (0.1–0.4 nA) evoked graded voltage changes that were less than those in the normal mice (Figure 3D). The differences in graded voltage changes (0.3 nA: 25.89 mV; 0.4 nA: 31.9 mV) were even more than the resting membrane potential difference (18.56 mV) between the sOn αRGCs in the control and CUS mice when the injected currents were high, suggesting that high injected currents induced more depolarized membrane potentials in normal mouse sOn αRGCs. Different from the graded voltage, the spike numbers during current injection showed no differences between the recordings in control and CUS mice (Figure 3E). However, the injected high currents evoked significantly higher maximum spike rates in CUS mouse sOn αRGCs (Figure 3F), which might have resulted from their less depolarized membrane potentials with high injected currents, because much higher depolarization might have inactivated the spike generator in sOn αRGCs [41].

The passive electric properties can influence the spike features. We examined the CUS−induced alterations in spike waveform features by comparing the spike amplitude and the maximum rising slope between sOn αRGC in the control and CUS mice (Figure 3G). For sOn αRGCs recorded from the normal mouse retinas, the spike amplitude decreased with the increased injected currents (Figure 3H). In contrast, all spikes recorded from CUS mouse sOn αRGCs showed small spikes regardless of the injected currents, like those recorded in the normal mouse sOn αRGCs with high injected currents (Figure 3H). Accordingly, the spontaneously generated spikes recorded from the sOn αRGCs in the CUS group exhibited significantly lower amplitude (Figure 3H). However, the differences in spike amplitudes weakened significantly with increased injected currents. Examining the maximum rising slopes, the values were significantly different between the control and CUS groups only when there was no injection of current (Figure 3I).

**Figure 4:**
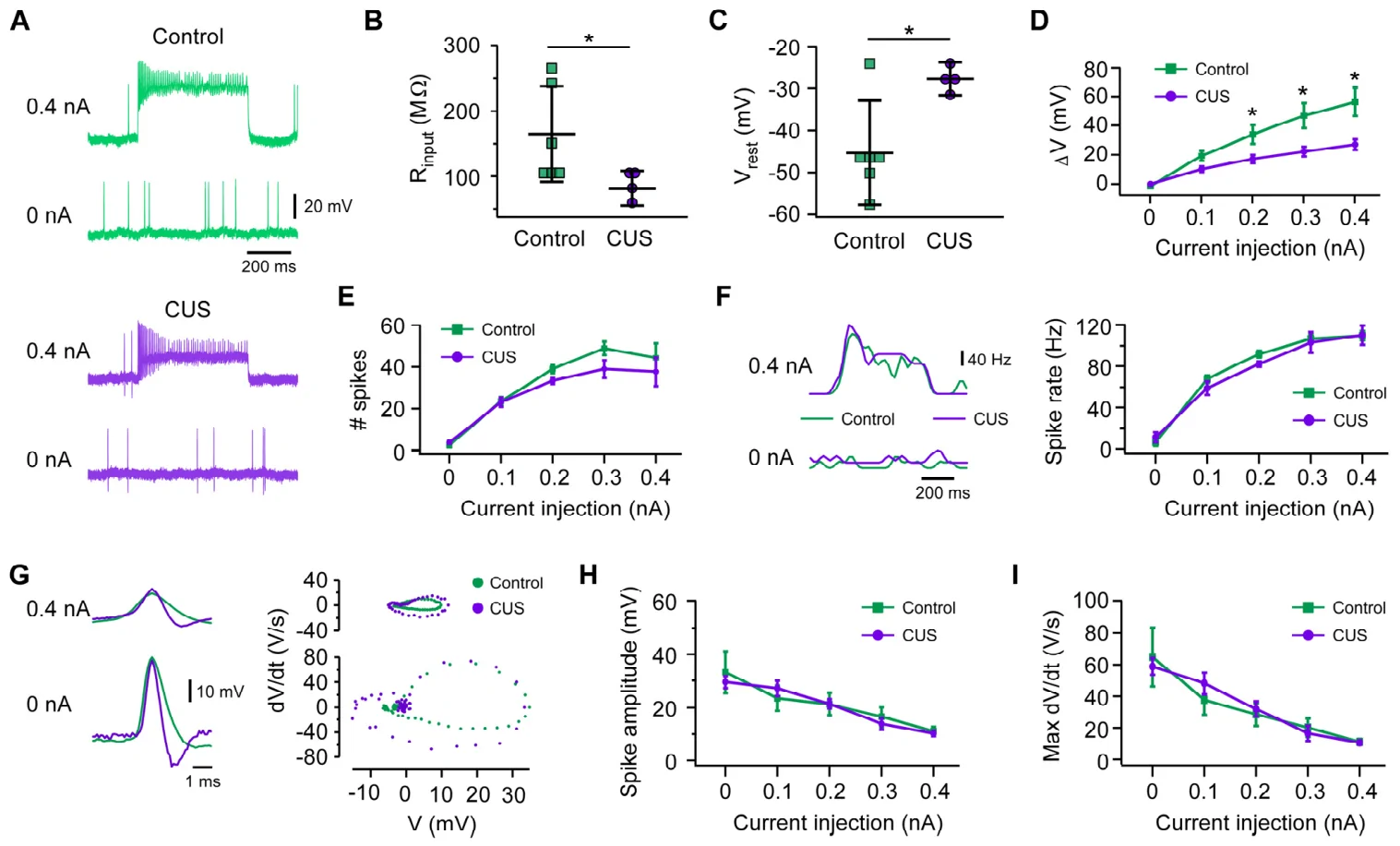
CUS induces intrinsic property alterations in tOff αRGCs. (**A**) Exemplary traces showing current injection evoked membrane potential changes for sOn αRGCs recorded from control (green) and CUS (purple) mouse retina in darkness. (**B**,**C**) Input resistance and resting membrane potential of tOff αRGCs recorded from normal mouse and CUS mouse retina. (**D**) The graded voltage responses of tOff αRGCs to different injected currents. (**E**) The number of spikes evoked by different injected currents. (**F**) Spike rate traces (**left**) calculated from traces in (**A**) and the maximum spike rate versus injected current (**right**). (**G**) Exemplary average spike waveforms (**left**) and their corresponding dV/dt plots (**right**). (**H**,**I**) The amplitude and maximum rising slope versus the injected currents. Control: n = 6 mice; CUS: n = 4 mice. Data are presented as mean ± SEM. * *p* < 0.05.

We next probed CUS−induced intrinsic property changes in tOff αRGCs (Figure 4A). Like sOn αRGCs, CUS induced lower membrane input resistance and higher resting membrane potentials in tOff αRGCs (Figure 4B,C), though the changes were relatively weaker than in sOn αRGCs. In parallel, the injected currents induced graded voltage increases in tOff αRGCs that also followed the sOn αRGCs changes, which were significantly lower than those recorded in the normal mouse retinas (Figure 4D). Meanwhile, unlike sOn αRGCs, tOff αRGCs showed no differences in their spike responses to current injection between the normal and CUS mice (Figure 4E,F). Regarding the spike waveform features, no matter whether for the spontaneously generated spikes or the current injection evoked spikes, no significant differences were detected regarding the spike amplitude and the maximum rising slopes of spikes between tOff αRGCs recorded from the control and CUS mouse retinas (Figure 4G–I), suggesting that the influence of CUS on the intrinsic properties of tOff αRGCs was weaker than on sOn αRGCs.

Together, these data indicate that CUS induced altered intrinsic properties of sOn αRGCs and tOff αRGCs, with lower input resistance and more depolarized resting membrane potentials, but more pronounced in sOn αRGCs. These passive electric property changes were associated with spike response changes and spike waveform changes that were observed in sOn αRGCs but not in tOff αRGCs, suggesting that, in mice with CUS−induced depression, intrinsic properties in sOn αRGCs showed more pronounced changes than tOff αRGCs, consistent with the greater CUS−induced cell loss in ipRGCs than in general RGCs.

### 3.4. Mice with CUS-Induced Depression Accompanied by Altered Light Responses in sOn and tOff αRGCs

As the intrinsic properties affected the RGC light responses, we examined whether and how the light−response properties of sOn and tOff αRGCs changed in the stressed mice. RGC light responses are stimulus−size−dependent due to their classical center−surround receptive fields (RFs). The RF center is classically defined as the retinal area where a small light spot evokes maximal neuronal excitation. In contrast, light stimuli presented in the regions surround the RF center typically evoke responses opposite to that evoked by the light stimuli presented in the center, a phenomenon known as the antagonistic surround. Here, we used two different sizes of light stimuli to evoke RGC responses. One was a small light spot (270 µm) that was close to the diameter of αRGC dendritic arbor, illuminating the excitation center of RGC receptive fields (RFs). The other one was full-field light (880 µm × 550 µm), which was larger than the αRGC dendritic arbor area and thus could illuminate both the excitation center and the antagonist surround of RFs. These two light stimuli allowed us to study not only the CUS−induced light response changes under light stimuli with different sizes, but also changes in the center–surround relationship.

**Figure 5:**
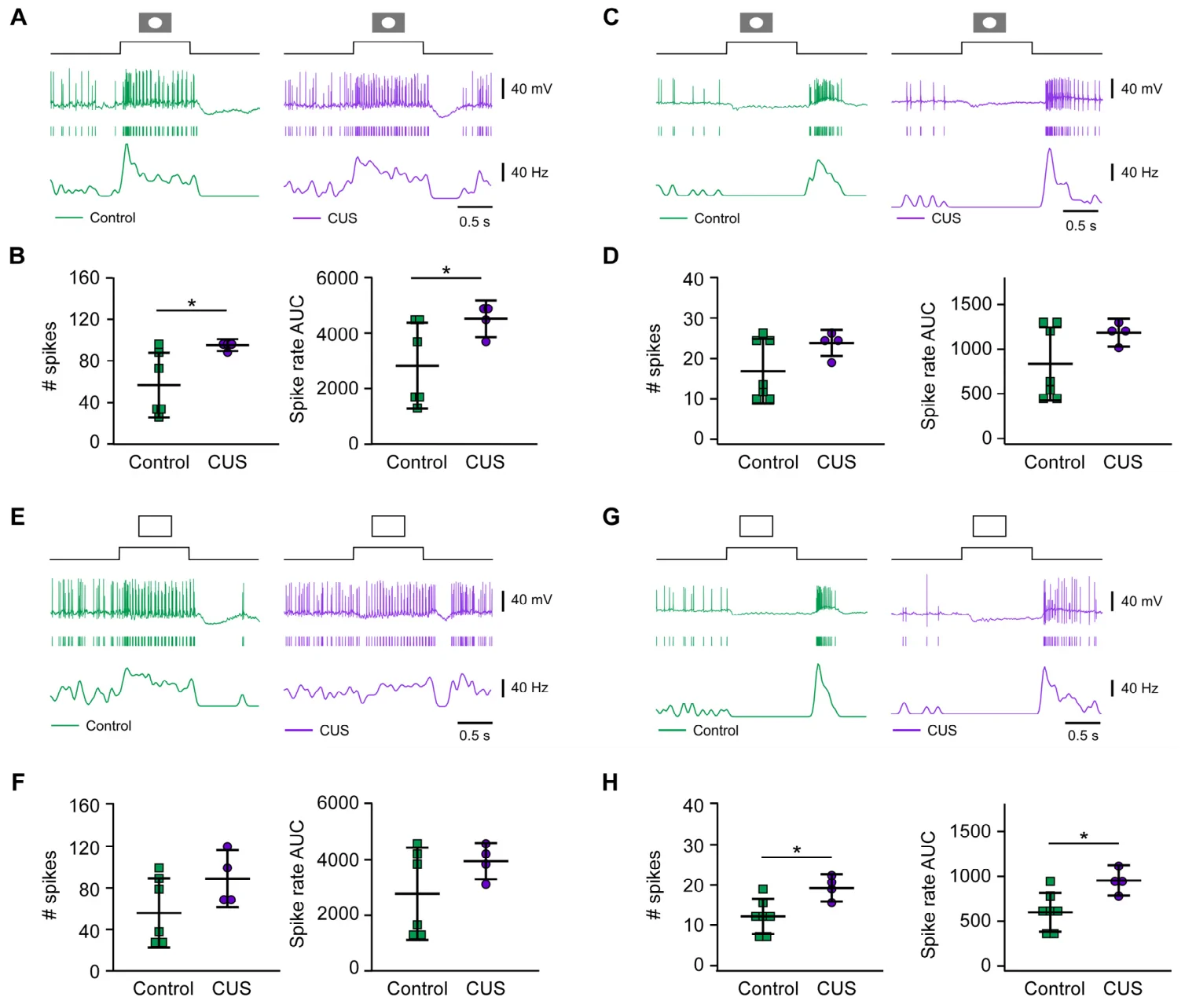
CUS induces light-response changes in sOn and tOff αRGCs. (**A**) Exemplary raw traces in response to small light spot and the corresponding binary spike train and spike rate traces. (**B**) The comparison of small light spot evoked spike numbers (**left**) and the calculated area under the spike rate curves (**right**) during light stimuli between sOn αRGCs recorded from normal mouse retinas and CUS mice retinas. Control: n = 6 mice; CUS: n = 4 mice. (**C**,**D**) Like (**A**,**B**), but for tOff αRGCs. Control: n = 7 mice; CUS: n = 4 mice. (**E**) Raw traces recorded from sOn αRGCs in response to full-field light stimuli and the corresponding binary spike train and spike rate traces. (**F**) Like (**B**), but for responses of sOn αRGCs to full-field light stimuli. Control: n = 6 mice; CUS: 4 mice. (**G**,**H**) Like (**E**,**F**), but for responses recorded from tOff αRGCs to full-field light stimuli. Control: n = 7 mice; CUS: n = 4 mice. Data are presented as mean ± SEM. * *p* < 0.05.

We found that the light responses of sOn αRGCs to a small light spot were significantly strengthened by chronic stress, with increased spike numbers and spike−rate AUC (Figure 5A,B). tOff αRGCs’ light responses to small light spots showed no significant differences between the control group and CUS group (Figure 5C,D). In contrast to the response changes observed under small light stimuli, full-field stimulation evoked no significant differences in sOn αRGCs, but did yield significant differences in tOff αRGCs between stressed and normal mouse retinas (Figure 5E–H). This different change in light responses to a small light spot and full−field light in sOn and tOff αRGCs suggests that CUS−induced depression is associated with changes in type−specific center–surround interactions.

## 4. Discussion

Dysfunction of RGCs and especially, ipRGCs, is closely related to the regulation of depressive disorders [16,17,18]. Thus, understanding depression-associated property changes in ipRGCs and general RGCs is crucial for both understanding the cellular basis of depression-related visual disturbances and developing effective treatments targeting the visual pathway. Unfortunately, the physiological and functional changes in RGCs are poorly understood. In this study, adopting a mouse model of depression induced by chronic stress, we assessed whether depression was associated with changes in retinal blood perfusion, which is critical for RGC survival and function. Then, we investigated how the properties of ipRGCs and general RGCs were influenced in depression, including their cell numbers, light responses, and intrinsic properties.

In depression, one of the most typical pathological phenotypes is the insufficient supply of blood flow, as described substantially in the brain [42,43,44]. The retina is a peripherally located part of the central nervous system. Theoretically, retinal blood perfusion may also change in depression, but there is little evidence about this. Our OCTA data showed that depression induced by CUS was associated with decreased blood perfusion, specifically in the retinal IPL. This layer-specific blood perfusion reduction might relate to the vertical distribution features of retinal blood perfusion. The retinal circulation is organized as a trilaminar angioarchitecture, comprising three distinct plexuses: the superficial, intermediate, and deep vascular layers (SVP, IVP, and DVP) [45]. The SVP is localized at the interface of the ganglion cell layer and the retinal nerve fiber layer, where it is formed by a combination of arterioles, venules, and capillaries. Compared to the SVP, the IVP and DVP are composed of more capillaries. The IVP is located at the junction of the inner nuclear layer and the inner plexiform layer (IPL), whereas the DVP resides within the outer plexiform layer, immediately adjacent to the synaptic terminals of the photoreceptors. Thus, the blood perfusion decrease in retinal IPL in depression suggests that retinal blood perfusion deficiency in depression occurs in the capillaries in the inner retina, consistent with capillary dysregulation observed in the brain [46,47,48,49].

Reduction of blood supply in the inner retina can induce damage to the inner retinal cells, including RGCs. In this study, we observed reduced RGC density in stressed mice, which presented less blood perfusion in the IPL. However, unlike cell loss under other pathological conditions with deficient blood supply, wherein ipRGCs often exhibit more resistance and with much less cell loss than the general RGC populations (e.g., [29,30,31]), here, in CUS-induced depression, ipRGCs showed more cell loss than the other RGCs. In addition to cell loss, our data suggest that the intrinsic electric properties of ipRGCs might also suffer more severe impairments than the general RGCs. While we did not directly study the intrinsic properties across different ipRGC types, our data showed that sOn αRGCs, known as M4 ipRGCs [50,51], showed more significant changes in input resistance, resting membrane potential, and spike waveform features compared with tOff αRGCs, which are not ipRGCs, suggesting that CUS induced more abnormal intrinsic properties in sOn αRGCs than tOff αRGCs. The greater susceptibility of ipRGCs to CUS is different from their resistance under many other pathologies that are also associated with deficient blood supply, such as glaucoma, retinal ischemia, and diabetic retinopathy (e.g., [29,30,31,32,52]), indicating that other mechanisms related to depression beyond hypoperfusion are involved in RGC damage in depression. The increased cell loss in ipRGC and greater intrinsic property changes in sOn αRGCs compared to tOff αRGCs after CUS support the common notion that ipRGCs are significantly affected in depression.

In addition to changes in RGC number and intrinsic properties, we found that CUS also modified their light responses in an illumination-area- and cell-type-dependent manner. In response to small light spots, sOn αRGCs—but not tOff αRGCs—exhibited increased responses. Conversely, under full-field stimulation, sOn αRGCs showed no differences between groups, whereas tOff αRGCs in the CUS group exhibited significantly stronger responses than controls. These opposing, size-dependent effects suggest distinct RGC-type-specific light response alterations following CUS. Collectively, these findings indicate that, in addition to type-specific intrinsic property changes, CUS drives divergent functional adaptations across RGC types, as exemplified by the differential responses of sOn and tOff αRGCs.

Regarding RGCs and other neurons, both the intrinsic properties and circuit modulations affect their functional outputs [53,54,55]. In CUS-induced depression, the increased depolarized resting membrane potential of sOn and tOff αRGCs is closer to the spike generation threshold potential and thus may make it easier to generate spikes. In line with this, the CUS group exhibited stronger spike responses than controls, specifically in sOn αRGC responses to small light spots and in tOff αRGC responses to full-field stimuli. However, sOn αRGCs showed no response differences when the surround was activated, nor did tOff αRGCs display any changes in their responses to small light spots. Thus, the light-response differences between the CUS and control groups were stimulus-size-dependent across both sOn and tOff αRGCs, suggesting modulation alterations at the retinal circuit level might be involved in CUS-induced depression. These results point to the complex alterations in both intrinsic properties and their functional circuit in depression. Thus, targeting only intrinsic properties or circuitry changes may not be enough to provide sufficient treatment for depression. Instead, alterations both in the intrinsic properties and at the circuit level should be considered.

Overall, this study shows that CUS-induced depression was associated with retinal pathology, with reduced blood flow perfusion in the IPL, decreased density of ipRGCs and general RGCs, and altered RGCs’ intrinsic properties and light responses. These findings suggest that distinct disturbances in retinal microcirculation and RGC function may serve as non-invasive biomarkers and actionable therapeutic targets for depression. However, the limitations of this study are obvious. First, the assessment of depression-like behavior relied exclusively on the FST and TST. Second, the mechanisms underlying the depression-associated reduction in blood perfusion and the RGC damage are not apparent, and these should be studied in the future. Third, while the intrinsic properties of RGCs may potentially affect their light responses, further studies should examine how the CUS-induced intrinsic properties of sOn αRGCs and tOff αRGCs relate to their light-response changes. Fourth, whether and how these CUS-induced impairments of RGCs affect visual behavior in living mice need to be investigated in the future. Finally, systematic investigations into the effects of varying durations of CUS exposure and post-CUS recovery timepoints on retinal pathology would be valuable.

## 5. Conclusions

Taken together, this study demonstrates that CUS-induced depression was accompanied by hypoperfusion in the retinal inner plexiform layer and type-specific pathological alterations in RGCs. The RGC pathological alterations included decreased cell density, especially for ipRGCs, as well as altered intrinsic properties and stimulus size-dependent changes in light-evoked responses. These findings deepen our understanding of depression-associated damage to RGCs.

## Data Availability

All data presented in this study is available at https://doi.org/10.5281/zenodo.20822064.

## References

[1] Ferrari A.J., Santomauro D.F., Aali A., Abate Y.H., Abbafati C., Abbastabar H., Abd ElHafeez S., Abdelmasseh M., Abd-Elsalam S., Abdollahi A. (2024). Global incidence, prevalence, years lived with disability (YLDs), disability-adjusted life-years (DALYs), and healthy life expectancy (HALE) for 371 diseases and injuries in 204 countries and territories and 811 subnational locations, 1990–2021: A systematic analysis for the Global Burden of Disease Study 2021. Lancet.

[2] Liu Q., He H., Yang J., Feng X., Zhao F., Lyu J. (2020). Changes in the global burden of depression from 1990 to 2017: Findings from the Global Burden of Disease study. J. Psychiatr. Res..

[3] Yuan Z., Quan J., Cui Y., Luo M. (2026). A circuit-based framework for depression: Reshaping the pathological attractor. Neuron.

[4] Tian H., Hu Z., Xu J., Wang C. (2022). The molecular pathophysiology of depression and the new therapeutics. MedComm.

[5] Marwaha S., Palmer E., Suppes T., Cons E., Young A.H., Upthegrove R. (2023). Novel and emerging treatments for major depression. Lancet.

[6] Davis A.K., Barrett F.S., May D.G., Cosimano M.P., Sepeda N.D., Johnson M.W., Finan P.H., Griffiths R.R. (2021). Effects of psilocybin-assisted therapy on major depressive disorder: A randomized clinical trial. JAMA Psychiatry.

[7] Hu H., Cui Y., Yang Y. (2020). Circuits and functions of the lateral habenula in health and in disease. Nat. Rev. Neurosci..

[8] Mahmud A., Karaman M.A. (2026). Disrupted synapses: Prefrontal cortex-reward circuit dysfunction in stress-induced depression-like behaviors. Front. Cell. Neurosci..

[9] Mannekote Thippaiah S., Wang M., Ransdell M., Dwivedi Y. (2025). The Clinical Impact of Habenular Dysfunction on Depression and Suicidality: A Literature Review and Discussion on the Implications for Psychiatric Practice. Curr. Behav. Neurosci. Rep..

[10] Zhang G.-M., Wu H.-Y., Cui W.-Q., Peng W. (2022). Multi-level variations of lateral habenula in depression: A comprehensive review of current evidence. Front. Psychiatry.

[11] Cho S.-E., Park C.-A., Na K.-S., Chung C., Ma H.-J., Kang C.-K., Kang S.-G. (2021). Left-right asymmetric and smaller right habenula volume in major depressive disorder on high-resolution 7-T magnetic resonance imaging. PLoS ONE.

[12] Logan R.W., Edgar N., Gillman A.G., Hoffman D., Zhu X., McClung C.A. (2015). Chronic stress induces brain region-specific alterations of molecular rhythms that correlate with depression-like behavior in mice. Biol. Psychiatry.

[13] Walker W.H., Walton J.C., DeVries A.C., Nelson R.J. (2020). Circadian rhythm disruption and mental health. Transl. Psychiatry.

[14] Crouse J.J., Carpenter J.S., Song Y.J.C., Hockey S.J., Naismith S.L., Grunstein R.R., Scott E.M., Merikangas K.R., Scott J., Hickie I.B. (2021). Circadian rhythm sleep–wake disturbances and depression in young people: Implications for prevention and early intervention. Lancet Psychiatry.

[15] Mendoza J. (2024). Circadian disruptions and brain clock dysregulation in mood disorders. Nat. Ment. Health.

[16] Mure L.S. (2021). Intrinsically photosensitive retinal ganglion cells of the human retina. Front. Neurol..

[17] Rao F., Xue T. (2024). Circadian-independent light regulation of mammalian metabolism. Nat. Metab..

[18] Yuan M., Liu H., Zhu R., Li Y., Song S., Yang A. (2025). Retinal light perception and biological rhythms: The role of light in sleep and mood from an ophthalmic perspective. Mol. Med. Rep..

[19] Baden T., Berens P., Franke K., Román Rosón M., Bethge M., Euler T. (2016). The functional diversity of retinal ganglion cells in the mouse. Nature.

[20] Bae J.A., Mu S., Kim J.S., Turner N.L., Tartavull I., Kemnitz N., Jordan C.S., Norton A.D., Silversmith W.M., Prentki R. (2018). Digital museum of retinal ganglion cells with dense anatomy and physiology. Cell.

[21] Lucas R.J. (2013). Mammalian inner retinal photoreception. Curr. Biol..

[22] Song X.M., Hu X.-W., Li Z., Gao Y., Ju X., Liu D.-Y., Wang Q.-N., Xue C., Cai Y.-C., Bai R. (2021). Reduction of higher-order occipital GABA and impaired visual perception in acute major depressive disorder. Mol. Psychiatry.

[23] Zhu M., Chen Y., Zheng J., Zhao P., Xia M., Tang Y., Wang F. (2025). Over-integration of visual network in major depressive disorder and its association with gene expression profiles. Transl. Psychiatry.

[24] Wu F., Lu Q., Kong Y., Zhang Z. (2023). A comprehensive overview of the role of visual cortex malfunction in depressive disorders: Opportunities and challenges. Neurosci. Bull..

[25] Zhang X., Wang Y., Xue S., Gong L., Yan J., Zheng Y., Yang X., Fan Y., Han K., Chen Y. (2024). Chronic stress in adulthood results in microvascular dysfunction and subsequent depressive-like behavior. Sci. Rep..

[26] Fang X., Jiang S., Wang J., Bai Y., Sub C., David K., Neal B., Lu Y.L.X. (2021). Chronic unpredictable stress induces depression-related behaviors by suppressing AgRP neuron activity. Mol. Psychiatry.

[27] Troubat R., Barone P., Leman S., Desmidt T., Cressant A., Atanasova B., Brizard B., El W., Surget A., Belzung C. (2021). Neuroinflammation and depression: A review. Eur. J. Neurosci..

[28] Lucassen P.J., Pruessner J., Sousa N., Almeida O.F.X., Marie A., Rajkowska G., Swaab D.F., Czéh B. (2014). Neuropathology of stress. Acta Neuropathol..

[29] Li S., Yang C., Zhang L., Gao X., Wang X., Liu W., Wang Y., Jiang S., Wong Y.H., Zhang Y. (2016). Promoting axon regeneration in the adult CNS by modulation of the melanopsin/GPCR signaling. Proc. Natl. Acad. Sci. USA.

[30] Zhang N., He X., Xing Y., Yang N. (2022). Differential susceptibility of retinal ganglion cell subtypes against neurodegenerative diseases. Graefe’s Arch. Clin. Exp. Ophthalmol..

[31] La Morgia C., Ross-Cisneros F.N., Sadun A.A., Hannibal J., Munarini A., Mantovani V., Barboni P., Cantalupo G., Tozer K.R., Sancisi E. (2010). Melanopsin retinal ganglion cells are resistant to neurodegeneration in mitochondrial optic neuropathies. Brain.

[32] Zhu M., Wu Y., Gao H., Qi F., Zhang X., Ran Y. (2025). Differential regulation of mTOR activity in retinal ganglion cells underlies their distinct susceptibility to ischemia/reperfusion. Commun. Biol..

[33] Ma Q., Zhou J., Yang Z., Xue Y., Xie X., Li T., Yang Y. (2022). Mingmu Xiaoyao granules regulate the PI3K/Akt/mTOR signaling pathway to reduce anxiety and depression and reverse retinal abnormalities in rats. Front. Pharmacol..

[34] Gubin D., Neroev V., Malishevskaya T., Kolomeichuk S., Cornelissen G., Yuzhakova N., Vlasova A., Weinert D. (2023). Depression scores are associated with retinal ganglion cells loss. J. Affect. Disord..

[35] Gao H., Ding Z., Xu B., Wu Y., Zhu M., Zhang X., Qi F., Liu J., Huo M., Li S. (2026). Chronic stress disrupts microglial-vascular interaction via AT1R signaling and altered microglial state to drive depression-like behaviors. Acta Neuropathol. Commun..

[36] Ferguson L.R., Dominguez J.M., Balaiya S., Grover S., Chalam K. (2013). V Retinal Thickness Normative Data in Wild-Type Mice Using Customized Miniature SD-OCT. PLoS ONE.

[37] Zhao B., Ni Y., Zhang H., Zhao Y., Li L. (2022). Endothelial deletion of TBK1 contributes to BRB dysfunction via CXCR4 phosphorylation suppression. Cell Death Discov..

[38] Warwick R.A., Kaushansky N., Sarid N., Golan A., Rivlin-Etzion M. (2018). Inhomogeneous encoding of the visual field in the mouse retina. Curr. Biol..

[39] Oesterle J., Ran Y., Stahr P., Kerr J.N.D., Schubert T., Berens P., Euler T. (2025). Task-specific regional circuit adaptations in distinct mouse retinal ganglion cells. Sci. Adv..

[40] Baden T., Euler T., Berens P. (2020). Understanding the retinal basis of vision across species. Nat. Rev. Neurosci..

[41] Chang L., Ran Y., Yang M., Auferkorte O., Butz E., Hüser L., Haverkamp S., Euler T., Schubert T. (2024). Spike desensitisation as a mechanism for high-contrast selectivity in retinal ganglion cells. Front. Cell. Neurosci..

[42] Cooper C.M., Chin Fatt C.R., Liu P., Grannemann B.D., Carmody T., Almeida J.R.C., Deckersbach T., Fava M., Kurian B.T., Malchow A.L. (2020). Discovery and replication of cerebral blood flow differences in major depressive disorder. Mol. Psychiatry.

[43] Chiappelli J., Adhikari B.M., Kvarta M.D., Bruce H.A., Goldwaser E.L., Ma Y., Chen S., Ament S., Shuldiner A.R., Mitchell B.D. (2023). Depression, stress and regional cerebral blood flow. J. Cereb. Blood Flow Metab..

[44] Briggs R., Carey D., Claffey P., McNicholas T., Newman L., Nolan H., Kennelly S.P., Kenny R.A. (2019). The association between frontal lobe perfusion and depressive symptoms in later life. Br. J. Psychiatry.

[45] Grimes W.N., Berson D.M., Sabnis A., Hoon M., Sinha R., Tian H., Diamond J.S. (2025). Layer-specific anatomical and physiological features of the retina’s neurovascular unit. Curr. Biol..

[46] Matsuno H., Tsuchimine S., O’Hashi K., Sakai K., Hattori K., Hidese S., Nakajima S., Chiba S., Yoshimura A., Fukuzato N. (2022). Association between vascular endothelial growth factor-mediated blood–brain barrier dysfunction and stress-induced depression. Mol. Psychiatry.

[47] Yang X., Wang J., Zhang J., Zhang M., Hao A., Guo F., Huang X., Yan J., Zheng Y., Xia Y. (2025). Cerebrovascular-mediated dynamic alterations in neurovascular coupling: A key pathological mechanism of depression. Cell Biosci..

[48] Sinka L., Kovari E., Santos M., Herrmann F.R., Gold G., Hof P.R., Bouras C., Giannakopoulos P. (2012). Microvascular changes in late-life schizophrenia and mood disorders: Stereological assessment of capillary diameters in anterior cingulate cortex. Neuropathol. Appl. Neurobiol..

[49] Mills W.A., Savory N.A., Lopez-Ortiz A.O., Lentferink D.H., González Ibáñez F., Agochi P., Rastegar E., Gupta A., Gupta D., Suram A. (2025). Microglial cyclooxygenase-1 modulates cerebral capillary basal tone in vivo in mice. Nat. Commun..

[50] Estevez M.E., Fogerson P.M., Ilardi M.C., Borghuis B.G., Chan E., Weng S., Auferkorte O.N., Demb J.B., Berson D.M. (2012). Form and function of the M4 cell, an intrinsically photosensitive retinal ganglion cell type contributing to geniculocortical vision. J. Neurosci..

[51] Kinder L., Lindner M. (2025). Expression of osteopontin in M2 and M4 intrinsically photosensitive retinal ganglion cells in the mouse retina. Investig. Ophthalmol. Vis. Sci..

[52] Mills S.A., Jobling A.I., Dixon M.A., Bui B.V., Vessey K.A., Phipps J.A. (2021). Fractalkine-induced microglial vasoregulation occurs within the retina and is altered early in diabetic retinopathy. Proc. Natl. Acad. Sci. USA.

[53] Ran Y., Huang Z., Baden T., Schubert T., Baayen H., Berens P., Franke K., Euler T. (2020). Type-specific dendritic integration in mouse retinal ganglion cells. Nat. Commun..

[54] Stuart G.J., Spruston N. (2015). Dendritic integration: 60 years of progress. Nat. Neurosci..

[55] Diamond J.S. (2017). Inhibitory interneurons in the retina: Types, circuitry, and function. Annu. Rev. Vis. Sci..

